# Hemocompatibility Evaluation of Thai *Bombyx mori* Silk Fibroin and Its Improvement with Low Molecular Weight Heparin Immobilization

**DOI:** 10.3390/polym14142943

**Published:** 2022-07-20

**Authors:** Tanrada Fungmongkonsatean, Jirapas Jongjitwimol, Pussadee Paensuwan, Teonchit Nuamchit, Duangduan Siriwittayawan, Sorada Kanokpanont, Siriporn Damrongsakkul, Piyanuch Thitiwuthikiat

**Affiliations:** 1 Graduate Program in Biomedical Sciences, Faculty of Allied Health Sciences, Naresuan University, Phitsanulok 65000, Thailand; tanrada.f@hotmail.com; 2 Department of Medical Technology, Faculty of Allied Health Sciences, Naresuan University, Phitsanulok 65000, Thailand; jirapasj@nu.ac.th; 3 Department of Optometry, Faculty of Allied Health Sciences, Naresuan University, Phitsanulok 65000, Thailand; pussadeep@nu.ac.th; 4 Department of Cardio-Thoracic Technology, Faculty of Allied Health Sciences, Naresuan University, Phitsanulok 65000, Thailand; teonchitn@nu.ac.th (T.N.); duangduans@nu.ac.th (D.S.); 5 Department of Chemical Engineering, Faculty of Engineering, Chulalongkorn University, Bangkok 10330, Thailand; sorada.k@chula.ac.th (S.K.); siriporn.d@chula.ac.th (S.D.); 6 Biomaterial Engineering for Medical and Health Research Unit, Chulalongkorn University, Bangkok 10330, Thailand

**Keywords:** Thai silk fibroin, hemocompatibility, low molecular weight heparin, fibroblast growth factor-2

## Abstract

*Bombyx mori* silk fibroin (SF), from Nangnoi Srisaket 1 Thai strain, has shown potential for various biomedical applications such as wound dressing, a vascular patch, bone substitutes, and controlled release systems. The hemocompatibility of this SF is one of the important characteristics that have impacts on such applications. In this study, the hemocompatibility of Thai SF was investigated and its improvement by low molecular weight heparin (LMWH) immobilization was demonstrated. Endothelial cell proliferation on the SF and LMWH immobilized SF (Hep/SF) samples with or without fibroblast growth factor-2 (FGF-2) was also evaluated. According to hemocompatibility evaluation, Thai SF did not accelerate clotting time, excess stimulate complement and leukocyte activation, and was considered a non-hemolysis material compared to the negative control PTFE sheet. Platelet adhesion of SF film was comparable to that of the PTFE sheet. For hemocompatibility enhancement, LMWH was immobilized successfully and could improve the surface hydrophilicity of SF films. The Hep/SF films demonstrated prolonged clotting time and slightly lower complement and leukocyte activation. However, the Hep/SF films could not suppress platelet adhesion. The Hep/SF films demonstrated endothelial cell proliferation enhancement, particularly with FGF-2 addition. This study provides fundamental information for the further development of Thai SF as a hemocompatible biomaterial.

## 1. Introduction

Cardiovascular diseases (CVDs) are still the leading causes of death globally [1]. CVDs, such as coronary heart disease, cerebrovascular disease, and peripheral arterial disease, are generally caused by a blockage of blood vessels from fatty plaque (atherosclerosis). The gold standard treatment is a bypass graft surgery alongside medication and angioplasty. The best choice for graft surgery is autologous grafts due to the low risk of immune rejection; however, the choices of vascular grafts are limited [2]. Commercial synthetic grafts, such as polyethylene terephthalate (PET) and expanded polytetrafluoroethylene (ePTFE) which are non-degradable polymers [2], have been used in such cases. However, limited success is found, particularly in small diameter grafts (inner diameter smaller than 6 mm), due to poor patency rates. Thrombogenicity is the major problem of small prosthetic grafts due to the delay or lack of re-endothelialization [3]. Tissue engineering of vascular grafts has been widely explored in the last couple of decades as an alternative solution to bypass grafts. Naturally derived biodegradable materials have been studied and applied for small vascular grafts to improve their long-term patency and functionality. The ideal concept of biodegradable grafts is matching the material degradation rate to cellular ingrowth to form new native tissues with proper mechanical properties. 

Silk fibroin (SF), a natural fibrous protein from *Bombyx mori* (*B. mori*) silkworm cocoons, consists of heavy chains (390 kDa), light chains (26 kDa), and glycoproteins named P25 (30 kDa) [4]. The heavy chains consist of extended polypeptide chains linked by hydrogen bonds to form anti-parallel β-sheets serving as the main structure of SF. From its structure, SF is very stable and provides excellent mechanical properties [5]. SF shows biocompatibility and has been widely applied in various biomedical applications in research [6,7,8,9,10,11,12] and human clinical trials [13]. *B. mori* SF from the Thai strain, Nangnoi Srisaket 1, has been reported for its biocompatibility as a non- to slightly-irritant material compared to commercial products in in vivo experiments according to ISO10993-6 (Biological evaluation of medical devices—Part 6: Tests for local effects after implantation) [14], anti-inflammatory and hypoallergenic properties [15]. It has shown potential for various tissue engineering and drug delivery systems [16,17,18,19,20]. According to vascular tissue engineering, in our previous work, we fabricated a vascular patch prepared from Thai SF/gelatin lining with gelatin hydrogel, incorporating simvastatin-micelle. The patch successfully recruited endothelial progenitor cells and accelerated re-endothelialization [21]. 

To develop tissue-engineered vascular grafts, both cell compatibility and hemocompatibility are required. Hemocompatible materials cause no blood-related adverse events when the materials come into contact with blood, including platelet adhesion/activation, thrombogenicity, hemolysis, and inflammation. From a previous study, pure SF is not an active substrate for blood coagulation; however, it has low anti-thrombogenicity [22]. SF has been studied and its hemocompatibility has been improved in different ways [23,24,25,26]. Although the structural components of SF from different domesticated *B. mori* races, such as Thai, Japanese, and Chinese, are identical, differences have been reported regarding amino acid content [27] and crystallinity [28]. Regarding amino acid content, the hydrophobic group of Thai SF (82%) has been reported to be higher than that of Japanese (77%) and Chinese (77%) SF. Thai SF also showed a higher water contact angle than that of the other two SF [27]. Relating to hemocompatibility, a hydrophobic surface has a higher tendency for plasma protein and platelet adsorption than a hydrophilic surface [29]. However, based on our knowledge, there are no reports on the hemocompatibility of Thai SF. In this work, the hemocompatibility of Thai SF, Nangnoi Srisaket 1 with human blood components was determined based on ISO standard 10993-4 (Biological evaluation of medical devices—Part 4: Selection of tests for interactions with blood), including coagulation, platelet adhesion, hemolysis, and inflammation in terms of complement and leukocyte activation. The results of improving hemocompatibility and biocompatibility of SF using low molecular weight heparin (LMWH) and fibroblast growth factor-2 (FGF-2) immobilization were also demonstrated. The findings provide fundamental knowledge of Thai SF, which has previously been proven to have the potential to be successfully applied in various biomedical applications and could be beneficial for future development and application as a blood-contacting biomaterial, especially for vascular graft tissue engineering.

## 2. Materials and Methods

### 2.1. Preparation of Silk Fibroin (SF) Solution

Thai *Bombyx mori* silk cocoons, Nangnoi-Srisaket 1, were kindly provided by the Queen Sirikit Sericulture Center, Nakhonratchasima province, Thailand. All reagents used in this study were purchased from Sigma-Aldrich, St. Louis, MO, USA, unless otherwise stated. SF solution was prepared following a method previously established [16]. Silk cocoons were boiled in 0.02 M sodium carbonate solution for 20 min and rinsed with deionized (DI) water to remove silk sericin. The boiling steps were repeated twice and air-dried at room temperature to obtain SF fibers. The SF fibers were dissolved in 9.3 M lithium bromide (LiBr) solution at 60 °C for 4 h. The solution was then dialyzed against DI water using cellulose membrane tubes (molecular weight cut-off at 12–16 kDa, Viskase Companies, Inc., Willowbrook, IL, USA) for 2 d to remove the LiBr. The solution was then centrifuged twice at 4000 rpm at 4 °C for 20 min to remove impurities. The concentration of the solution was approximately 6 %wt. The SF solution was stored at 4 °C before use. 

### 2.2. Preparation of Immobilized Heparin with SF (Hep/SF) Films

Enoxaparin sodium (Clexane®, Sanofi-Aventis, Paris, France) which is a LMWH was used to immobilize with SF solution. N′-ethylcarbodiimide hydrochloride/N-Hydroxysuccinimide (EDC/NHS) were used as chemical crosslinkers. Carboxylic acid groups of heparin were activated by mixing with 0.4 M of EDC and 0.24 M of NHS in 0.05 M of 2-(N-Morpholino) ethanesulfonic acid (MES buffer, pH 5.6) [30]. Heparin was added to obtain the concentrations at 5, 10, and 15 mg/mL as suggested in previous reports for significantly prolonged blood coagulation and good cell compatibility [31,32,33]. After pre-activation at room temperature for 10 min, SF solution was added to the mixture to obtain the final concentration of SF at 4 %wt. The mixture was shaken at room temperature for 5 min and cast into polypropylene dishes and tissue culture plates before air drying overnight. After that, the films were washed twice with 0.05 M of MES buffer for 20 min each and DI water for 20 min before air drying. The sample nomenclature of the films was SF for SF films without heparin and 5, 10, and 15 Hep/SF for 5, 10, and 15 mg/mL heparin incorporated with SF films, respectively. 

### 2.3. Characterization

#### 2.3.1. Fourier Transform Infrared (FTIR) Spectroscopy

To evaluate the success of LMWH immobilization with SF films, FTIR spectroscopy in an attenuated total reflection (ATR) mode was performed. The samples were analyzed by using a FTIR spectrometer (PerkinElmer, Inc., Spokane, WA, USA) within the wavenumber range of 400–4000 cm^−1^ [34].

#### 2.3.2. Surface Charge Evaluation

To evaluate the surface charge of the polymeric solution, the zeta potential was measured. The SF and Hep/SF solution were diluted in phosphate-buffered saline (PBS) to obtain the final concentration of 0.5 %wt at pH 7.4. The zeta potential of the samples was measured by phase analysis light scattering using a Malvern Zetasizer Nano ZS (Malvern Instruments Ltd., Worcestershire, UK) at 25 °C [19].

#### 2.3.3. Surface Wettability Evaluation

To evaluate surface wettability, the water contact angle of the films was evaluated. The SF and Hep/SF films were prepared on circular glass slides. A PTFE film was also employed as a hydrophobic reference material. Water contact angles were determined after dropping 5 µL of DI water on the films for 10 s at room temperature using an optical contact angle measuring instrument (OCA 20, DataPhysics Instruments GmbH, Filderstadt, Germany).

#### 2.3.4. In Vitro Heparin Release Profile Analysis

The SF and Hep/SF films were cut into 2 cm × 2 cm. The films were immersed in PBS containing 0.1 % w/v sodium azide (Ajax Finechem, Auckland, New Zealand) with or without 1 U/mL protease XIV. The films were incubated at 37 °C with water-bath shaking at 250 rpm. The buffer solution was collected and replenished periodically. To quantify released LMWH, a dimethylmethylene blue (DMMB) assay was performed. The absorbance at 525 nm was measured using a microplate reader (EnSpire® Multimode Plate Reader, PerkinElmer, Inc., Waltham, MA, USA) [35,36].

### 2.4. Hemocompatibility Evaluation

A hemocompatibility test was performed following test categories recommended in the standard ISO 10993-4, including coagulation, platelets, hematology, and complement system. The study was conducted in accordance with the Declaration of Helsinki. The protocol was approved by the Human Research Ethics Committee of Naresuan University Institutional Review Board (IRB No. 836/58) and Buddhachinaraj Phitsanulok Hospital Institutional Review Board (IRB No. 143/60). Human blood components were provided by Buddhachinaraj Phitsanulok Hospital, Phitsanulok, Thailand. 

#### 2.4.1. In Vitro Coagulation Test

The SF, Hep/SF films, and PTFE sheets were pre-incubated under PBS at 37 °C for 5 min. The PBS was replaced with fresh frozen plasma (FFP) that was already thawed at 37 °C and incubated at 37 °C for 1 h. After that, activated partial thromboplastin time (APTT), prothrombin time (PT), and thrombin time (TT) were measured with Dade Actin FS Activated PTT Reagent, Thromborel^®^ S Reagent, and Test Thrombin Reagent, respectively, following the manufacturer’s protocols (Siemens Healthcare Diagnostics, Marburg, Germany). The clotting time was read and recorded using a semi-automated blood coagulation analyzer (Sysmex^®^ CA-50, Sysmex Corporation, Kobe, Japan). The experiments were performed in triplicate for each sample.

#### 2.4.2. Platelet Adhesion and Activation

The SF and Hep/SF solutions were coated on glass slides and placed in 24-well tissue culture plates. A PTFE sheet was used as a negative control. The samples were pre-incubated with PBS at 37 °C for 5 min. Platelet concentrate (PC) at the concentration of 5.5 × 10^10^ platelets/mL was diluted with PBS to obtain the concentration of 2.5 × 10^8^ platelets/mL. The PBS was then replaced with diluted PC and incubated at 37 °C for 1 h. After incubation, the samples were rinsed twice with PBS to wash out non-adhered platelets. To quantify the number of adhered platelets, a lactate dehydrogenase (LDH) assay (Thermo Scientific, Rockford, IL, USA) was performed following the manufacturer’s instructions. The absorbance at 490 nm was measured using the microplate reader [37]. The concentrated PC was serial diluted and used as a standard. In a parallel experiment, the activation of platelets was evaluated by their morphological changes. The adhered platelets on the samples were fixed with 2.5% glutaraldehyde in PBS and kept at 4 °C. The samples were then dehydrated by immersing in ethanol at a serial concentration of 30%, 50%, 70%, 95%, and 100% for 10 min for each step. The samples were dried by the critical point dry technique using an automated critical point dryer (Leica EM CPD300, Leica Microsystems, Wetzlar, Germany) and sputter coated with gold. The number of the adhered platelets and their morphological changes were recorded using a scanning electron microscope (SEM, JSM-IT500HR, JEOL, Tokyo, Japan) (magnification 1500x) [38]. 

#### 2.4.3. Complement and Leukocyte Activation

To determine complement and leukocyte activation, an enzyme-linked immunosorbent assay (ELISA) was performed. The SF, Hep/SF films, and PTFE sheets were pre-incubated with PBS at 37 °C for 5 min. The PBS was replaced with lithium heparinized blood and incubated at 37 °C for 1 h. Blood performed accordingly without samples was used as a control. After incubation, the plasma was isolated by centrifugation at 1400× *g* for 15 min. Complement activation was determined by using ELISA Kits for complement fragments C3a (ab279352, Abcam, Cambridge, UK) and C5a (ab193695, Abcam, Cambridge, UK). Leukocyte activation was determined by using ELISA Kits for polymorphonuclear neutrophil (PMN) elastase (ab119553, Abcam, Cambridge, UK). Measurements were performed according to the manufacturer’s instructions and in quadruplicate for each sample. The absorbance was measured using a microplate reader (SpectraMax® iD3, Molecular Devices, LLC., San Jose, CA, USA) [39,40].

#### 2.4.4. Hemolysis Test

The SF, Hep/SF films, and PTFE sheets with 1 × 1 cm^2^ were prepared. Packed red cells (PRC) were pre-incubated at 37 °C for 30 min and diluted with normal saline at the ratio of 1:1. The samples were pre-incubated with PBS at 37 °C for 5 min. The PBS was replaced with diluted blood and incubated at 37 °C for 1 h. Normal saline and distilled water were used as negative and positive controls, respectively. After incubation, the blood was centrifuged at 2500 rpm, 4 °C for 5 min. Supernatants containing red blood cell lysate were collected. To measure released hemoglobin, the absorbance at 545 nm of the supernatants was measured using the microplate reader. The percentage of hemolysis was calculated according to the following equation [41].

$$Percentage\;of\;hemolysis\;\left(\%\right)=\frac{OD_{Sample}-OD_{Negative\;control}}{OD_{Positive\;control}-OD_{Negative\;control}}$$

where OD is optical density.

### 2.5. Evaluation of EA.hy926 Proliferation

The SF and Hep/SF solutions were coated in 48-well tissue culture plates and air-dried at room temperature. The samples were sterilized with ethylene oxide gas. Human umbilical vein endothelial cell line, EA.hy926, purchased from the American Type Culture Collection (ATCC^®^ CRL-2922™, Manassas, VA, USA), was pre-cultured in Dulbecco’s Modified Eagle Medium (DMEM) supplemented with 10% fetal bovine serum and 1% Penicillin-Streptomycin. The media and supplements were purchased from Gibco, Grand Island, NY, USA. The samples were pre-adsorbed with 10 ng/mL of human fibroblast growth factor-2 (FGF-2) (R&D Systems, Minneapolis, MN, USA) in PBS at 4 °C overnight. The wells were then washed twice with PBS. The cells at a density of 5000 cells/well were seeded and cultured at 37 °C and 5% CO_2_. Cells cultured on a tissue culture-treated plate (TCP) were used as a control. After 1, 3, and 5 days of culture, the number of cells was measured by using a 3-[4,5-dimethylthiazol-2-yl]-2,5 diphenyl tetrazolium bromide (MTT, VWR Life Science, Solon, OH, USA) assay. MTT solution was added to each well to obtain a final concentration of 0.5 mg/mL and incubated at 37 °C for 1.5 h. After incubation, dimethyl sulfoxide (DMSO) was used to dissolve purple formazan crystals. The absorbance at 570 nm of the solution was measured using the microplate reader [42]. 

### 2.6. Statistical Analysis

Data were reported as mean ± SD. For statistical analysis, one-way analysis of variance (ANOVA) with Bonferroni post hoc test was used to evaluate the differences among data sets using IBM^®^ SPSS^®^ Statistics software version 17 under the license of Naresuan University. A *p*-value of ˂ 0.05 was considered statistically significant.

## 3. Results and Discussion

### 3.1. Characterization

#### 3.1.1. ATR-FTIR Analysis

**Figure 1:**
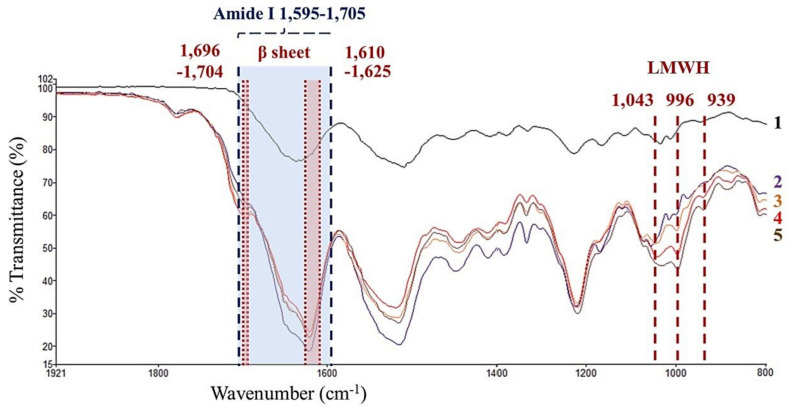
FTIR spectra of the samples including (1) non-crosslinked SF, (2) crosslinked SF, (3) 5, (4) 10, and (5) 15 Hep/SF films, respectively. The secondary structures of LMWH (peaks at 939, 996, and 1043 cm^−1^), amide I (region 1595–1705 cm^−1^), and β-sheet (regions 1696–1704 and 1610–1625 cm^−1^) are shown. The data was presented in Thai Proceedings of the Graduate Research Conference (GRC) 2018, Thailand. Reprinted with permission from ref. [47]. Copyright 2022 Oraphan Anurukvorakun.

ATR-FTIR was performed to investigate the success in the immobilization of LMWH to SF films. Figure 1 shows the FTIR spectra of the samples including non-crosslinked SF film, crosslinked SF film, 5, 10, and 15 Hep/SF films. Compared to non-crosslinked SF film, crosslinked SF film and all Hep/SF films show the absorption peaks of amide I region 1595–1705 cm^−1^ and β-sheet regions 1610–1625 and 1696–1704 cm^−1^ (Figure 1, spectra 2–5). EDC/NHS was used to crosslink between a carboxyl group and an amine group of SF by covalent bonds, resulting in the conformation transition from random coil or α-helix to β-sheet and the formation of amide bonds within the SF [43]. For all Hep/SF films, the secondary structures of LMWH are shown in the peaks at 939, 996, and 1043 cm^−1^ (Figure 1, spectra 3–5). LMWH is polysulfated glycosaminoglycans derived from unfractionated heparin via chemical or enzymatic depolymerization [44]. According to the Hep/SF spectra, the peak at 1043 cm^−1^ could be attributed to the vibration of S═O symmetric stretching groups, while the peaks at 939 and 996 cm^−1^ could be assigned to C–O–S stretches [45,46]. The results indicated that LMWH was successfully immobilized with SF films.

#### 3.1.2. Surface Charge

The surface charge of the SF and Hep/SF particles in PBS solution was evaluated by zeta potential measurement. Table 1 shows the zeta potential values of the SF and Hep/SF solutions at pH 7.4. The SF solution showed a negative charge (−4.01 ± 0.09 mV). The Hep/SF solutions tended to be more negative with increasing heparin concentrations. 

The hydrophobic domain is the main structure of SF consisting of glycine, alanine, serine, and tyrosine, which are neutral amino acids. However, there are small hydrophilic regions including N- and C- termini and hydrophilic spacers in the hydrophobic domain. The hydrophilic regions of SF consist of both charged and uncharged amino acids. Among charged amino acids (4.11% by mole), there are more negatively charged amino acids (2.78% by mole), aspartic acid and glutamic acid, than positively charged amino acids (1.33% by mole) [27]. A previous study reported that the isoelectric point (pI) of SF was at pI 4–5 [48]. Similarly, the isoelectric point of Thai SF, Nangoi Srisaket 1, was at pI 4 [49], resulting in the negative surface charge expression of SF at pH 7.4. Enoxaparin, the LMWH used in this study, is a linear polysaccharide with a highly negative charge composed of repeating disaccharide units of D-glucosamine and uronic acid linked by 1→4 glycosidic bonds [50]. The surface charge of Hep/SF particles which were a combination of both negatively charged SF and enoxaparin was then expressed as a negative charge in PBS.

#### 3.1.3. Surface Wettability

The surface wettability of the SF and Hep/SF films was evaluated by water contact angle measurement. Table 2 shows the water contact angles of the SF and Hep/SF films and PTFE sheet. PTFE sheet, a hydrophobic material, showed the highest water contact angle at 105.70 ± 1.18°. The water contact angle of the SF film was 60.35 ± 0.38°. Water contact angles of Hep/SF films were lower than that of the SF film and were decreased with increasing heparin concentrations. The immobilization of LMWH, a sulfated glycosaminoglycan containing hydrophilic groups such as sulfonic acid, carboxyl acid, and hydroxyls, to hydrophobic SF improved its hydrophilicity [46]. 

#### 3.1.4. In Vitro Heparin Release Profiles

**Figure 2:**
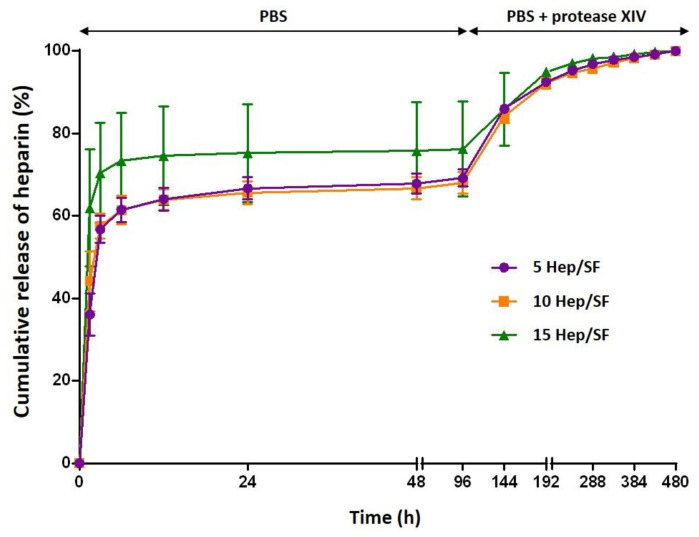
Percentages of cumulative release profiles of LMWH from (−ο−) 5, (−□−) 10, and (−Δ−) 15 Hep/SF films under PBS and PBS containing protease XIV.

Figure 2 shows the percentages of cumulative release profiles of LMWH from the Hep/SF films. Under a physiological buffer (PBS, pH 7.4, 37 °C), there was a rapid burst release of heparin during the first 6 h and then a continuously slow release was observed until a stable level was reached from 24 to 96 h (4 days). Percentages of heparin cumulative release of 5, 10, and 15 Hep/SF films at 96 h under PBS were 69.15 ± 2.06, 68.01 ± 2.61, and 76.22 ± 11.55%, respectively. The initial burst release of heparin was possibly from the diffusion of non-crosslinked heparin adsorbed on the film surface. After 96 h, the buffer was replaced with PBS containing 1 U/mL of protease XIV. The cumulative release of heparin was increased with the gradual degradation of the films. The Hep/SF films were completely degraded at 480 h (20 days). In the presence of the enzyme, the release of heparin would result from the degradation of the films as the sudden changes in the release profiles were observed after enzyme addition. 

SF, a natural fibrous protein, is a biodegradable material that can be slowly degraded in vitro and in vivo in response to proteolytic enzymes. Therefore, in vitro heparin release analysis was performed to experimentally demonstrate the release mechanism of heparin from the SF films. From the result, the release mechanism of heparin was related to the diffusion and enzymatic degradation of the films, which can be tailored by the crosslinking process.

### 3.2. Hemocompatibility Evaluation

#### 3.2.1. In Vitro Coagulation Test

**Figure 3:**
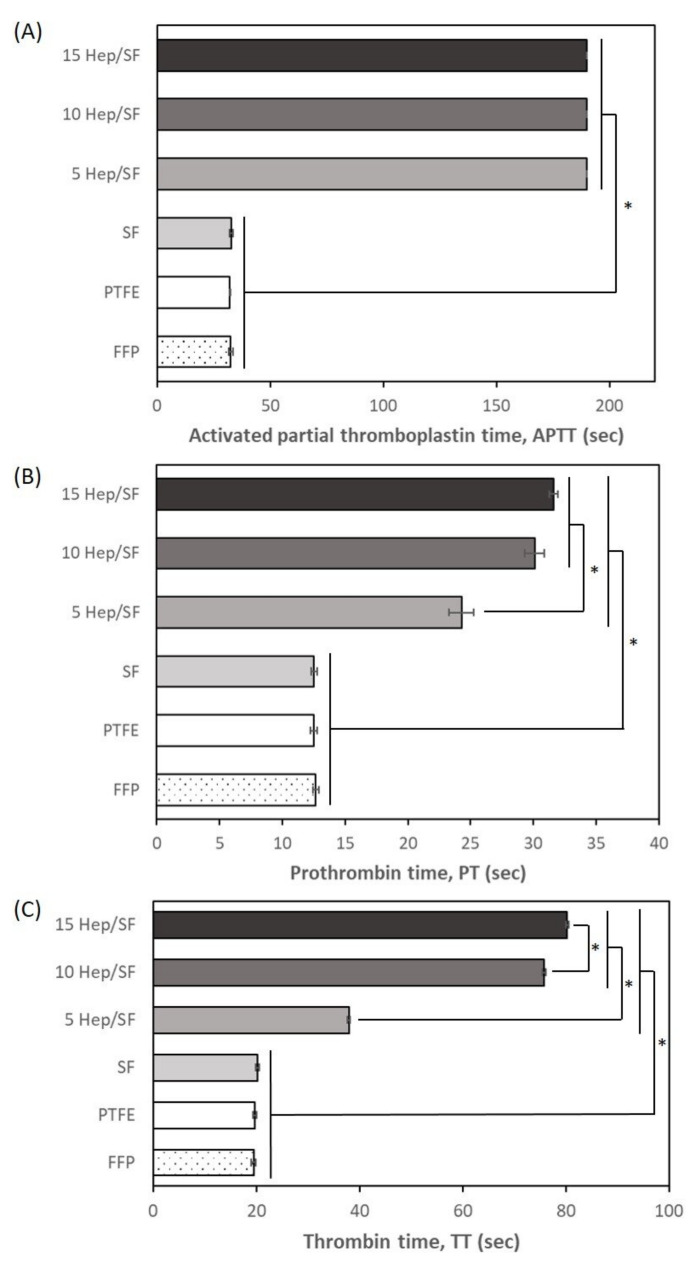
In vitro coagulation times including (**A**) activated partial thromboplastin time, APTT, (**B**) prothrombin time, PT, and (**C**) thrombin time, TT, of SF and Hep/SF films and PTFE sheet after 1 h of incubation. * *p* < 0.05 between the groups.

To evaluate the effect of the materials on the activation of blood coagulation, the sample films were incubated with FFP at 37 °C for 1h and blood coagulation tests were performed. The blood coagulation cascade can be divided into three pathways including intrinsic (contact activation), extrinsic (tissue factor), and common pathways, leading to fibrin formation [51]. Therefore, in vitro blood coagulation tests were performed to evaluate all three pathways, including APTT for intrinsic and common pathways, PT for extrinsic and common pathways, and TT for common pathways [52]. Figure 3A–C show results of coagulation times of the SF and Hep/SF films including APTT, PT, and TT, respectively. SF had comparable coagulation times to the FFP and PTFE. APTT, PT, and TT values of 5, 10, and 15 Hep/SF films were significantly prolonged compared to those of the SF film, PTFE sheet, and FFP (*p* < 0.05). APTT values of 5, 10, and 15 Hep/SF films in this study were at 190 s, which was the maximum limit of the measuring apparatus. Compared to 5 Hep/SF films, PT and TT values of 10 and 15 Hep/SF films were significantly longer (*p* < 0.05). TT value of 15 Hep/SF film was significantly longer than that of the 10 Hep/SF film (*p* < 0.05). From the results, coagulation times of the Hep/SF films would be increased with increasing heparin concentration. 

Regarding surface-mediated reactions, once blood contacts the materials, the plasma proteins are the first to be adsorbed to the surface of the materials which is a foreign body. The deformation of the adsorbed proteins, including proteins of intrinsic clotting factors (factor XII), leads to the activation of the intrinsic pathway. Negatively charged surfaces, such as glass and cellulose, and also hydrophilic and hydrophobic surfaces were reported to trigger the activation of factor XII [53]. The results of comparable coagulation times of the SF and FFP indicated that Thai SF film was not a thrombogenic material, which agreed with a previous report [54]. LMWH is an anticoagulant that binds and activates antithrombin III (AT-III), leading to the inactivation of several clotting enzymes, including thrombin, FXIIa, FXIa, FIXa, FXa, and FV, to prevent thrombus formation [55]. Therefore, the immobilization of LMWH to Thai SF films could improve the antithrombogenic property, which was consistent with previous reports [45,56].

#### 3.2.2. Platelet Adhesion

**Figure 4:**
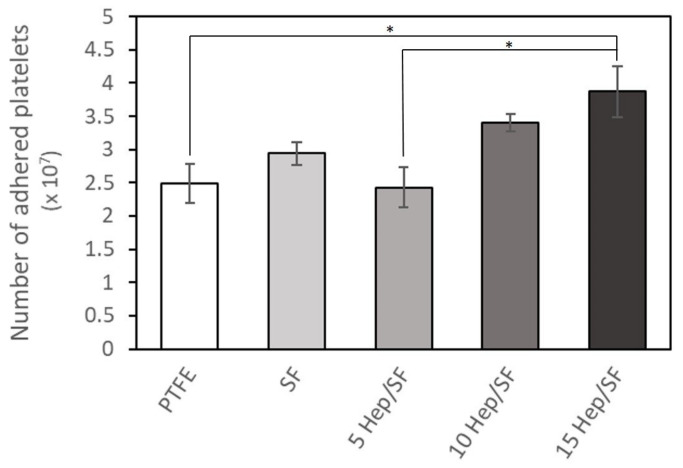
The number of adhered platelets on the samples after 1 h of incubation under diluted platelet concentrate and determined by LDH assay. * *p* < 0.05 between the groups.

**Figure 5:**
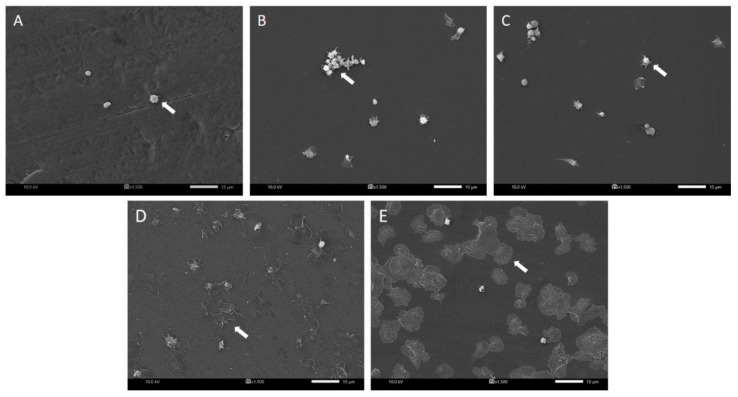
SEM images of adhered platelets on the samples including (**A**) PTFE, (**B**) SF, (**C**) 5, (**D**) 10, and (**E**) 15 Hep/SF films. White arrows indicate adhered platelets. Images were taken at the magnification of 1500X. Scale bars: 10 μm.

Figure 4 shows the number of adhered platelets on the SF, Hep/SF films, and PTFE samples measured by LDH assay. There was no significant difference between the number of adhered platelets on SF film, PTFE sheet, and 5 and 10 Hep/SF films. However, the number of adhered platelets was significantly increased in 15 Hep/SF films compared to that of 5 Hep/SF films and PTFE sheets (*p* < 0.05). Figure 5 shows the morphology and activation states of adhered platelets on the samples under SEM at 1500X magnification. The morphological changes of adhered platelets were generally divided into 5 groups, including discoid, dendritic, spread dendritic, spread, and fully spread depending on the stage of activation [57]. The majority of adhered platelets on SF, PTFE sheet, and 5 Hep/SF films were dendritic-shaped platelets (Figure 5A–C), while the majority of the adhered platelets on 10 Hep/SF films were both spread dendritic and spread (Figure 5D). Fully spread-shaped platelets were mostly observed on the 15 Hep/SF film (Figure 5E). 

A platelet adhesion test is often used to determine the hemocompatibility of materials because platelet adhesion and activation are an important process of thrombus formation [57]. Regarding hydrophilic/hydrophobic (wettability) of surfaces, with the absence of plasma proteins, the platelets directly adhered to the surfaces. The adhered platelets increased gradually as the surface wettability increased and they were also more activated. On the other hand, with the presence of plasma proteins, the platelets were more adhered and activated on the more hydrophobic surface [58]. Normally, blood-biomaterial contact induces platelet adhesion and activation via plasma protein adsorption. Plasma proteins are adsorbed on biomaterial surfaces and interact with glycoprotein receptors on platelets. Afterward, the platelets are adhered on the surface and activated leading to their morphological changes. The activated platelets release alpha and dense granule contents; for example, adenosine diphosphate and thromboxane A2, which promote the adhesion, activation, and aggregation of circulating platelets. Platelets are aggregated by fibrinogen and von Willebrand factor crosslinking with glycoprotein receptors between platelets [57,59]. In general, the more hydrophilic surface, which plasma proteins tend to bind to in a lower amount and less affinity compared to the more hydrophobic surface, tends to have lower protein adsorption and platelet adhesion, leading to more blood compatibility [60]. However, in this study, although the number of adhered platelets on 5 and 10 Hep/SF films was not significantly different from that on SF and PTFE, the Hep/SF films could not suppress interaction with platelets. This was consistent with a previous report [24]. As plasma proteins play important roles in platelet adhesion, plasma protein adsorption on the films needs to be further investigated.

#### 3.2.3. Complement and Leukocyte Activation

**Figure 6:**
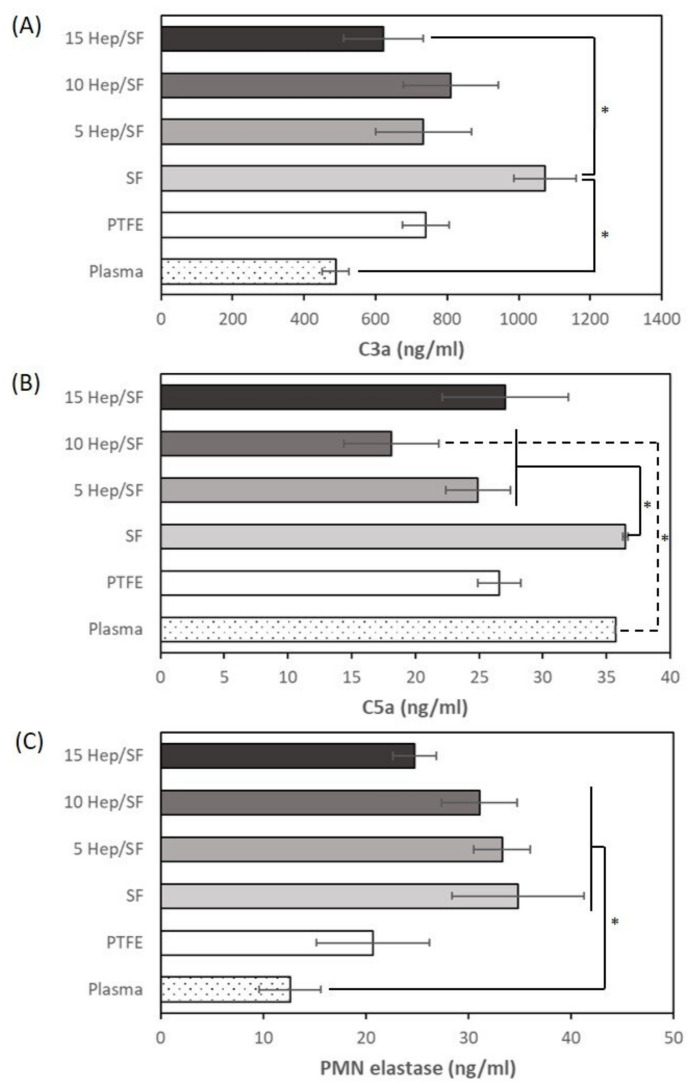
Complement and leukocyte activation of the samples including (**A**) C3a, (**B**) C5a, and (**C**) PMN elastase, respectively, after incubation of the samples under blood for 1h. * *p* < 0.05 between the groups.

The inflammatory response of the SF and Hep/SF films was investigated via the activation of complement and leukocyte. Figure 6A,B shows the results of complement activation including C3a and C5a, respectively, after incubation of the samples under blood for 1 h. C3a of the SF film was not significantly different from that of PTFE. However, C3a concentration of the SF film was significantly higher than that of plasma and the 15 Hep/SF film. C3a concentrations of 5, 10, and 15 Hep/SF films were not significantly different from those of plasma and PTFE. For C5a, in general, C5a concentrations of the samples were comparable to that of plasma. C5a concentration of 10 Hep/SF film was significantly lower than that of plasma. According to leukocyte activation, PMN elastase was evaluated (Figure 6C). PMN elastase of 15 Hep/SF film was not significantly different from that of plasma and PTFE. However, PMN elastase concentrations of all samples were not more than that of healthy people (43.7 ± 5.4 ng/mL) [61]. 

Complement system is a part of the human innate immune response that defends against pathogens by their direct lysis or by promoting phagocytosis via leukocytes. Thus, the complement system is related to leukocyte recruitment (chemotaxis) and activation [62]. Complement proteins circulate in the blood as inactive forms and become active when they interact with pathogens or foreign surface structures. Complement factors are activated in an enzyme cascade through three pathways, including the classic complement pathway, the alternative pathway, and the lectin pathway [59]. In the context of hemocompatibility evaluation, C3a and C5a complement protein fragments generated in the cascade and PMN elastase released from activated leucocytes are recommended for determination. C3a and C5a are potent anaphylatoxins that promote inflammation [59]. The hydrophobic surface was reported to induce complement activation more than the hydrophilic surface [63]. From the results, in general, the SF and Hep/SF films did not exaggerate the activation of complement and leukocytes, which related to previous reports [24,64]. However, LMWH immobilization could be beneficial in terms of increasing hydrophilicity.

#### 3.2.4. Hemolysis Test

**Figure 7:**
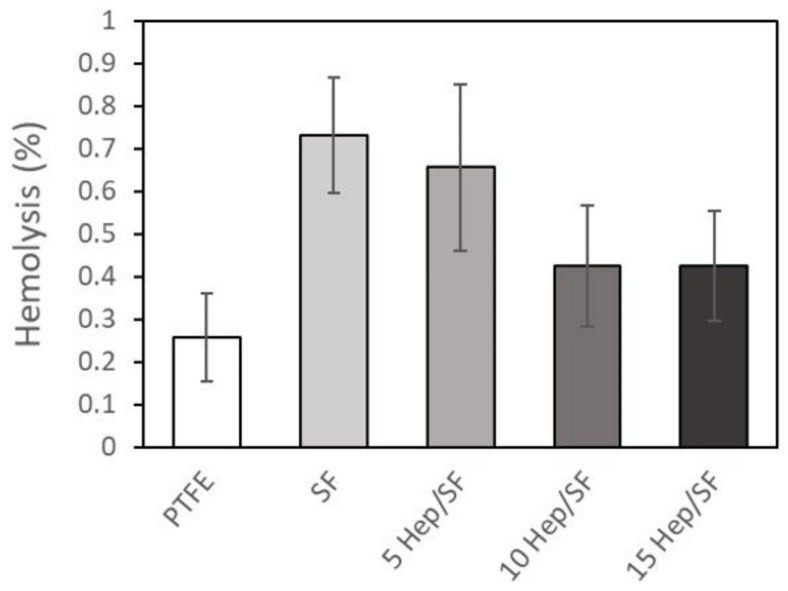
Percentages of hemolysis induced by the samples after 1 h of incubation under diluted blood. Percentages of hemolysis were calculated by comparing to those of normal saline and distilled water as negative and positive controls, respectively.

The purpose of the hemolysis test was to investigate the effect of the SF and Hep/SF films on red blood cell rupture. Figure 7 shows the percentages of hemolysis of the SF, Hep/SF films, and PTFE sheet. There was no significant difference in hemolysis between these samples. Overall, the percentages of hemolysis in all samples did not reach 1%. 

Hemolysis is influenced by various factors such as chemical toxicity, air–blood interfaces, and shear force [53]. Chemical toxicity from the materials themselves or the unclean procedure would affect red blood cell hemolysis. According to the American Society for Testing and Materials (ASTM) standard ASTM F756-00 (Standard Practice for Assessment of Hemolytic Properties of Materials), a percentage of hemolysis less than 2% is considered non-hemolysis [65]. Our results indicated that the films contain no chemical reagents that are toxic to red blood cells and could be considered non-hemolytic materials, which is consistent with previous reports [46,66].

### 3.3. Evaluation of EA.hy926 Proliferation

**Figure 8:**
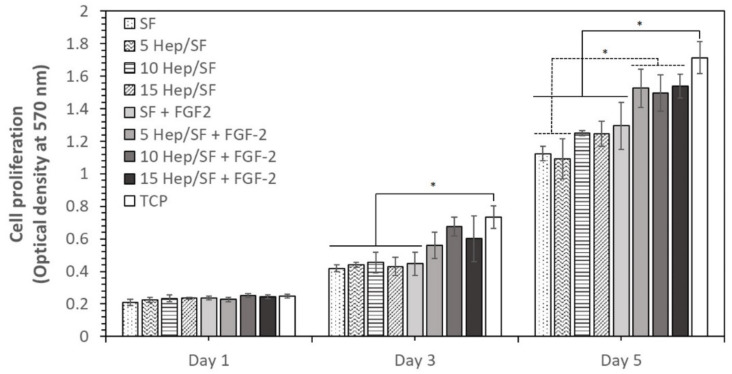
Proliferation of EA.hy926 endothelial cell line cultured on various SF-based samples and control (tissue culture-treated plate, TCP). * *p* < 0.05 between the groups.

To evaluate the bioactivity of immobilized heparin and cell compatibility, EA.hy926 endothelial cell proliferation on the SF and Hep/SF films with or without FGF-2 was determined (Figure 8). It was noticed that the cells proliferated continuously over 5 days of culture. There was no significant difference in cell attachment and growth between all samples on day 1. After 3 and 5 days, cell growth on 5, 10, and 15 Hep/SF films with FGF-2 was not significantly different from that on TCP. On day 5, cell growth on 5, 10, and 15 Hep/SF films with FGF-2 was significantly higher than that on SF and 5 Hep/SF without FGF-2. 

In normal physiology, the endothelial layer acts as a blood-tissue interface. Healthy endothelial cells are non-thrombogenic. They maintain hemostasis by regulating inflammation, permeability, thrombosis, and fibrinolysis [67]. Therefore, endothelialization is considered one of the strategies for improving hemocompatibility and prolonging the vascular graft patency rate [68]. LMWH can control the release of growth factors such as vascular endothelial growth factor (VEGF) and FGF-2 by electrostatic interaction. The negatively charged sulfate groups of heparin interact with positively charged growth factors [69]. FGF-2 is a potent angiogenic molecule that stimulates endothelial cell proliferation, migration, and angiogenesis [70]. FGF-2 also accelerates wound healing and tissue repair [71]. The interactions of FGF-2 with other endogenous molecules such as heparin improve its stability [69]. From the results, cell proliferation on the SF films was significantly lower than that of the Hep/SF with FGF-2. The structure of *B. mori* SF, which mainly consists of a crystalline domain expressing a predominantly hydrophobic surface and also lacks an amino acid RGD (arginyl-glycyl-aspartic acid) sequence, one of the cell adhesion recognition motifs, would affect less support cell attachment and proliferation [72]. Therefore, LMWH immobilization with FGF-2 growth factors demonstrated the cell compatibility improvement of *B. mori* SF. The results were in agreement with previous reports on cell proliferation enhancement of heparin/FGF-2 [73,74].

The hemocompatibility of materials is affected by various material properties such as surface chemistry, surface texture, surface wettability, surface charge, porosity, and surface modulus [75]. Thrombus formation involves the activation of complex interconnected regulatory systems including coagulation cascade, complement cascade, and cellular components of the blood, such as leukocytes and platelets [68]. Therefore, to further develop hemocompatible materials, in-depth hemocompatibility testing both in vitro and in vivo covering interconnected systems leading to thrombosis is required.

## 4. Conclusions

In this study, the hemocompatibility of Thai *B. mori* SF films was investigated and its improvement by LMWH immobilization was demonstrated. According to the hemocompatibility evaluation, Thai SF did not accelerate clotting time, excess stimulate complement and leukocyte activation, and was considered a non-hemolysis material compared to the negative control PTFE sheet. Platelet adhesion of the SF film was also comparable to that of the PTFE sheet. For hemocompatibility enhancement, we successfully immobilized LMWH with SF films, which improved the surface hydrophilicity of the SF films. The release of heparin from Hep/SF films could be sustained over 4 days in PBS via diffusion and was gradually enhanced via enzymatic degradation with protease XIV over 20 days. The Hep/SF films demonstrated prolonged clotting time and slightly lower complement and leukocyte activation. However, the Hep/SF films could not suppress platelet adhesion. The Hep/SF films demonstrated the enhancement of endothelial cell proliferation, particularly with FGF-2 addition. This is the first report on the hemocompatibility of Thai domestic *B. mori*, Nangnoi Srisaket 1, SF, which has the potential to be applied in various biomedical applications. This work provides fundamental information for the further development of Thai SF as a hemocompatible biomaterial.

## Figures and Tables

**Table 1:** Zeta potential of silk fibroin (SF) and heparin-silk fibroin (Hep/SF) solutions.

Samples	Zeta Potential (mV)
SF solution	−4.01 ± 0.09
5 Hep/SF solution	−4.21 ± 0.07
10 Hep/SF solution	−4.46 ± 0.15
15 Hep/SF solution	−4.64 ± 0.28

**Note:** The data was presented in Thai Proceedings of the Graduate Research Conference (GRC) 2018, Thailand [47].

**Table 2:** Water contact angles of silk fibroin (SF) and heparin-silk fibroin (Hep/SF) films compared to PTFE.

Samples	Water Contact Angles	
	Degree (°)	Images
PTFE sheets	105.70 ± 1.18	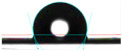
SF films	60.35 ± 0.38	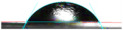
5 Hep/SF films	47.80 ± 3.60	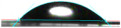
10 Hep/SF films	40.60 ± 2.44	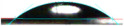
15 Hep/SF films	37.20 ± 2.59	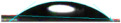

**Note:** The data was presented in Thai Proceedings of the Graduate Research Conference (GRC) 2018, Thailand [47].

## Data Availability

Data is contained within the article.
